# Large-scale single-neuron speech sound encoding across the depth of human cortex

**DOI:** 10.1038/s41586-023-06839-2

**Published:** 2023-12-13

**Authors:** Matthew K. Leonard, Laura Gwilliams, Kristin K. Sellers, Jason E. Chung, Duo Xu, Gavin Mischler, Nima Mesgarani, Marleen Welkenhuysen, Barundeb Dutta, Edward F. Chang

**Affiliations:** 1 Department of Neurological Surgery, University of California, San Francisco, San Francisco, CA USA; 2 Weill Institute for Neurosciences, University of California, San Francisco, San Francisco, CA USA; 3 Mortimer B. Zuckerman Mind Brain Behavior Institute, Columbia University, New York, NY USA; 4 Department of Electrical Engineering, Columbia University, New York, NY USA; 5 IMEC, Leuven, Belgium

**Keywords:** Cortex, Language

## Abstract

Understanding the neural basis of speech perception requires that we study the human brain both at the scale of the fundamental computational unit of neurons and in their organization across the depth of cortex. Here we used high-density Neuropixels arrays^1–3^ to record from 685 neurons across cortical layers at nine sites in a high-level auditory region that is critical for speech, the superior temporal gyrus^4,5^, while participants listened to spoken sentences. Single neurons encoded a wide range of speech sound cues, including features of consonants and vowels, relative vocal pitch, onsets, amplitude envelope and sequence statistics. Neurons at each cross-laminar recording exhibited dominant tuning to a primary speech feature while also containing a substantial proportion of neurons that encoded other features contributing to heterogeneous selectivity. Spatially, neurons at similar cortical depths tended to encode similar speech features. Activity across all cortical layers was predictive of high-frequency field potentials (electrocorticography), providing a neuronal origin for macroelectrode recordings from the cortical surface. Together, these results establish single-neuron tuning across the cortical laminae as an important dimension of speech encoding in human superior temporal gyrus.

## Abstract (web summary)

High-density single-neuron recordings show diverse tuning for acoustic and phonetic features across layers in human auditory speech cortex.

## Main

Speech perception is the process of transforming an acoustic signal into linguistic structures, such as syllables, words and sentences. The superior temporal gyrus (STG) is a critical area in the human brain for speech perception and comprehension^6–8^. Recent work with direct cortical surface field potential recordings (electrocorticography (ECoG)) has provided a window into how different sites across the surface of the gyrus are tuned to specific speech sounds, such as the features of consonants and vowels^9^, vocal pitch in prosody^10^ and syllabic cues in the speech envelope^11^. While this work has described speech encoding across the STG, a major limitation is that ECoG signals at each electrode reflect the combined activity of thousands of neurons. By contrast, established methods for recording single neurons using microelectrodes sample from only a small number of units. Therefore, neither is able to resolve the neuronal organization across the cortical depth.

To address the neuronal processing of speech in the human brain, we used high-density multielectrode Neuropixels probes^1–3^ to record cellular activity from hundreds of individual neurons across the cortical layers in STG while participants listened to naturally spoken sentences. This approach allowed us to address (1) what acoustic and phonetic speech features are encoded by single neurons; (2) the functional organization of neurons across cortical layers of STG^12–14^; and (3) how single-neuron activity relates to population activity recorded from the cortical surface using ECoG^15,16^. Understanding speech processing at the cellular level has the power to provide fundamental insights into the cortical representation of speech.

**Fig. 1:**
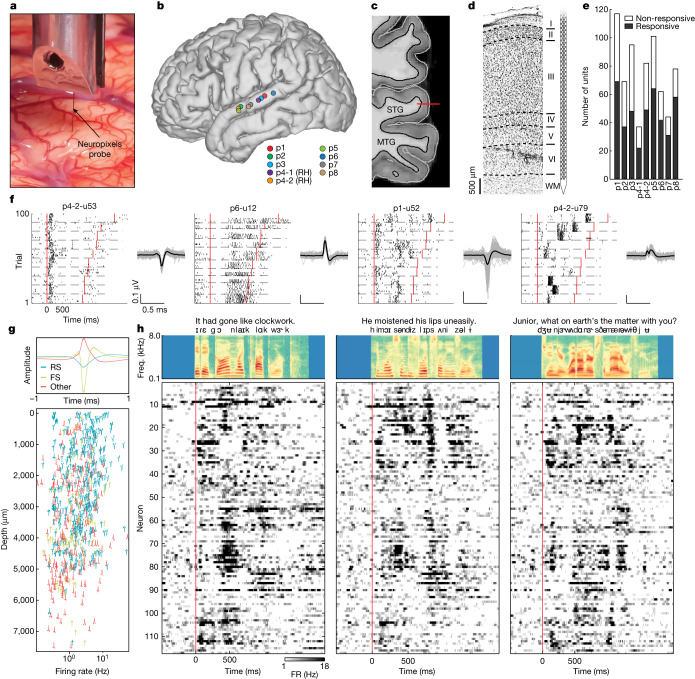
Large-scale human single-neuron recording across the cortical depth using Neuropixels probes. **a**, Close-up photograph of the Neuropixels probe inserted into the human cortex. **b**, Recording locations for nine penetrations (right STG sites (RH) plotted on the left hemisphere). **c**, Magnetic resonance imaging shows the approximate location of the Neuropixels probe spanning the full cortical depth in p1 (MTG, middle temporal gyrus). **d**, Histology from resected tissue at the insertion site in p1 provides approximate laminar boundaries within STG. **e**, Number of speech-responsive and non-responsive units. **f**, Single-trial spike rasters for example neurons showing how neurons respond differently to different sentences. Each neuron shows multiple trials of 10 different sentences (separated by dashed lines). Spike waveforms (mean and 100 randomly selected single spikes) are shown to the right. Red lines indicate sentence onset and offset. **g**, Three types of spike waveform (upper panel; FS, fast spiking; RS, regular spiking) with distribution across the cortical depth in nine sites (lower panel). **h**, Thresholded PSTH (50 ms window) for three sentences (averaged across repetitions) from 117 neurons in p1 (sorted by depth) shows patterns of evoked activity across the depth. The upper panels show acoustic spectrograms of each sentence with word and phoneme annotations. freq., frequency; FR, firing rate.

**Extended Data Fig. 1:**
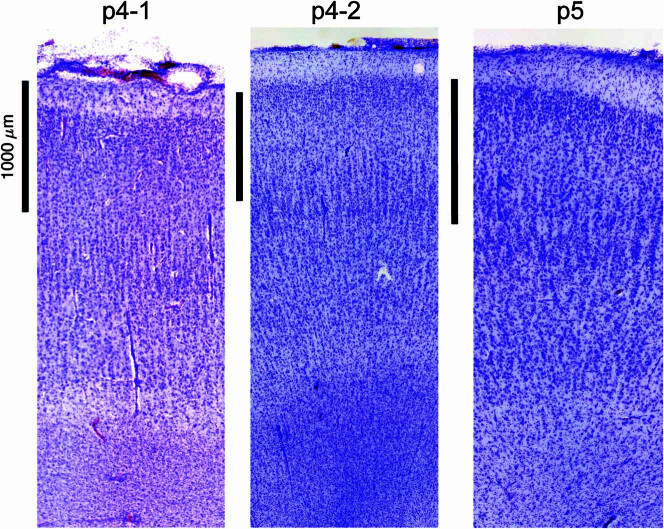
Histology from three additional recording sites. Each Nissl stain is from fixed tissue that was sectioned to cover the region immediately surrounding the Neuropixels insertion site. Attempts were made to localize the insertion site, but this was very difficult to do with standard anatomic pathology sampling. Therefore, we have provided images from approximately the same area.

A Neuropixels probe^2^ was placed in the mid-posterior STG at nine locations in eight participants (seven left hemisphere, one right hemisphere) undergoing awake language mapping during neurosurgical procedures (Fig. 1a,b). The probe was inserted temporarily into tissue that was subsequently removed as part of temporal lobe epilepsy surgery or tumour resection^17^. The probe had 384 recording channels spanning 7.66 mm and was slowly inserted perpendicularly into the crown of the cortical gyrus to achieve a vertical orientation through the cortex (Fig. 1c). The perpendicular penetration allowed dense sampling of activity from neurons spanning the pial surface to the white matter boundary (Fig. 1c,d; Extended Data Fig. 1 shows the histology from other participants).

## Dense sampling of single-neuron spiking in STG

Eight participants were awake and listened to 200 naturally spoken sentences (produced by 103 unique male and female speakers), which span the natural variability in the acoustic, phonetic and prosodic aspects of English^18^. For visualization and model evaluation, 10 sentences were repeated 10 times, whereas the remaining 100 sentences were played once each (total experiment duration was 8.3 min) (‘Speech stimuli and procedures’). After performing automated spike sorting and manual curation^19^, we obtained 685 putative single units across all nine sites (*n* = 117, 69, 95, 37, 82, 101, 62, 44, 78 in each insertion). Of these units, 420 (61%) showed significant responses to the speech stimuli (Fig. 1e) (speech responsive is defined using the parameter-free ZETA test^20^ compared with silent periods).

When we aligned activity to speech onset for each of the 100 repeated trials, we observed a striking diversity of response patterns (Fig. 1f and Supplementary Video 1). For example, some neurons responded primarily at the start of sentences with either increased (p4-2-u53) or decreased (p6-u12) firing. Other units had highly specific increases in firing at different moments in each sentence (p1-u52, p4-2u79).

The large number of neurons we recorded enabled us to sample different putative cell types (for example, excitatory cells versus inhibitory interneurons) across the depth of the cortex. We clustered spike waveforms from the nine sites using *k*-means and found three distinct shapes: regular-spiking neurons, fast-spiking neurons^21,22^ and other neurons with broad positive peaks^23^ (Fig. 1g). Across the cortical depth, regular-spiking and positive-spiking neurons were the most prevalent, spanning all putative layers. Speech-evoked responses and speech feature encoding were largely similar across putative cell types (Supplementary Fig. 1). We did not find a relationship between response type (for example, enhanced versus suppressed firing) and putative cell types (Fig. 1g), and neurons with speech-evoked responses were represented by all waveform shapes (Fig. 1f and Supplementary Fig. 1). Fast-spiking neurons were not as common as regular-spiking and positive-spiking neurons, and they were found mostly in mid–deep layers.

The diversity of responses (Fig. 1f) and putative cell types (Fig. 1g) across the cortical depth suggests that, within a recording site perpendicular to the cortical surface, different cells are associated with distinct types of speech-evoked activity. We observed that within a site, neurons exhibited a wide variety of response patterns when presented with different spoken sentences and that these patterns varied as a function of cortical depth (Fig. 1h; Supplementary Fig. 2 shows a comparison between signal and noise correlations). Although we were unable to define the precise boundaries between layers (Methods), this large-scale picture of neuronal speech responses provides a highly detailed view of the response diversity for speech and suggests that STG neurons, even at a single location in the cortex, encode many different speech properties.

## Single-neuron responses to speech

**Fig. 2:**
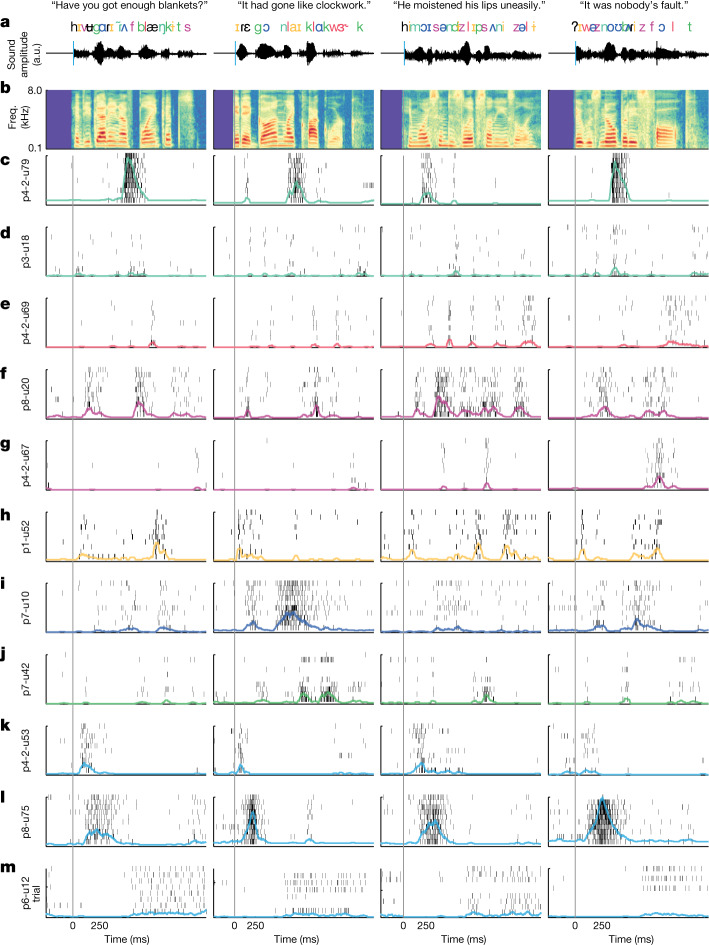
Single-trial rasters for example neurons show diversity of response types and tuning. **a**, Four example sentences with word- and phoneme-level transcriptions time aligned to the audio waveform. Phoneme/feature colours correspond to example units in **c**–**m**, which were labelled by hand for visualization purposes. **b**, Acoustic spectrogram of speech stimuli. Rasters for each neuron and sentence. Rows correspond to the number of repeats for that neuron and sentence. Coloured lines are the smoothed (50 ms window) PSTHs across trials. **c**,**d**, Two examples of neurons responding primarily to nasal sounds (for example, /m/, /n/). Note that even similarly tuned neurons can have very different spiking properties (for example, primarily bursting (p4-2-u79) versus sparse firing (p3-u18)). **e**, Neuron responding primarily to approximant sounds (for example, /l/, /r/, /w/). **f**,**g**, Two examples of neurons that are selectively responsive to fricatives (for example, /s/, /z/, /f/). **h**, Neuron selectively responsive to high/front vowels (for example, /i/, /ɪ/). **i**, Neuron primarily responsive to low/back vowels (for example, /ɑ/, /ʌ/, /ɔ/). **j**, Neuron primarily responsive to plosives (for example, /b/, /d/, /g/, /p/, /t/, /k/). **k**–**m**, Neurons responsive to sentence onsets. Some units show increased firing at onset (**k**,**l**), whereas others show delayed firing (**m**). a.u., arbitrary unit.

Electrical stimulation mapping and neurophysiology data suggest that the STG is specialized for high-order, complex auditory speech processing^24–27^. To examine response selectivity to speech input, we first visualized single-trial activity for a wide variety of neurons across many different sentences and participants (Fig. 2). The purpose of this visualization was to provide a qualitative description of the raw data (single-neuron spikes). These example neurons demonstrate that activity was highly consistent across repeated presentations of the same sentence (Fig. 2a,b) and highly specific to particular speech cues (Fig. 2c–m and Supplementary Video 2)^28^.

In each recording, we found many neurons that responded to specific speech sounds (Fig. 2c–j). For example, we observed neurons that showed increased firing in response to nasal sounds, such as /m/ and /n/ (Fig. 2c,d). Some neurons responded specifically to approximant sounds, such as /l/, /w/ and /r/ (Fig. 2e). Others were tuned to fricative sounds, such as /s/, /z/, /f/ and /v/ (Fig. 2f,g). Some were tuned to high/front vowels, such as /i/ and /ɪ/ (Fig. 2h), and others were tuned to low/back vowels, such as /ɑ/, /ʌ/ and /ɔ/ (Fig. 2i). Finally, some neurons responded to plosive sounds, such as /b/, /d/, /g/, /p/, /t/ and /k/ (Fig. 2j). In each of these cases, responses were not specific to individual phonemes, but rather were selective to groups of speech sounds that share acoustic–phonetic features (the coloured phoneme labels shown in Fig. 2a). This suggests that tuning to speech sounds reflects auditory sensitivity to specific articulatory gestures during speaking (that is, voicing, plosive, nasal and so on)^29,30^ rather than individual phoneme consonants and vowels.

In addition, we also observed neurons with clear and highly specific responses to non-phonetic acoustic cues. For example, we found neurons that responded exclusively at the onset of sentences, regardless of the specific speech sounds (Fig. 2k,l)^31^. Others showed suppression with a characteristic period of no firing (Fig. 2m).

## Encoding variability within a cortical column

ECoG studies have revealed a spatial organization of speech feature representations across the surface of STG^4^. Yet, it remains unknown whether different neurons across a vertical column of cortex have homogeneous tuning (as seen in primary sensory cortices) or encode different speech properties. Furthermore, if neurons within a site are heterogeneous, it is unknown whether different representations cluster at particular depths, potentially reflecting the laminar structure of the cortex.

**Fig. 3:**
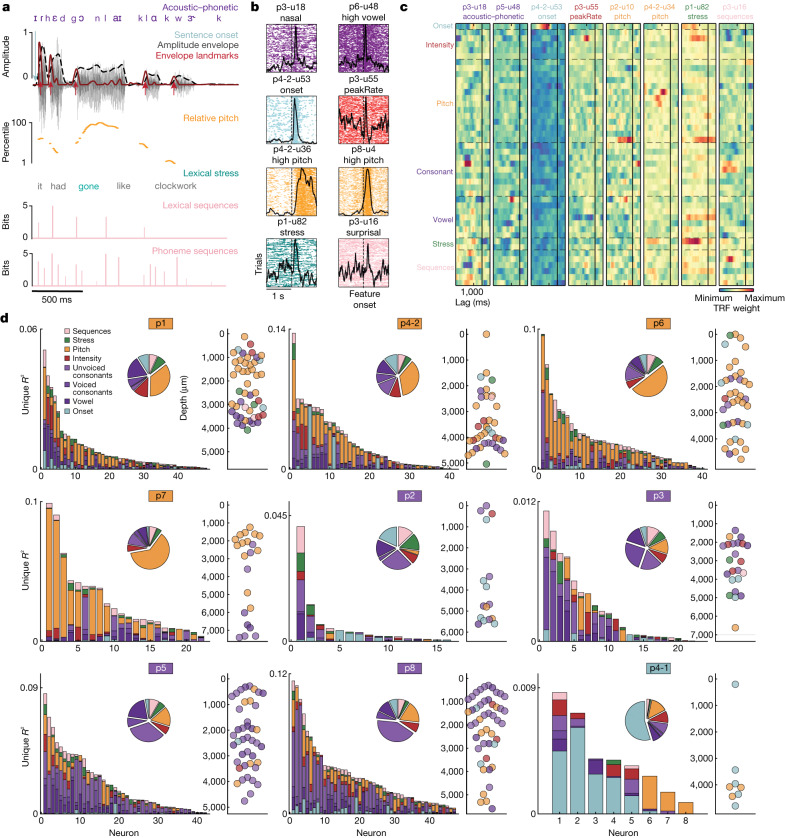
Encoding of heterogeneous speech features within and across cortical sites. **a**, Example sentence annotations with acoustic–phonetic (vowel, consonant), prosodic (relative pitch, intensity, stress, onset) and sequence statistics features. **b**, Spike rasters for eight neurons aligned to a subset of speech features. The *y* axis corresponds to all instances of the given feature (for example, all nasal sounds across all sentences). The *x* axis is aligned to the feature of interest (plus or minus 1 s). The black lines indicate the average response to all feature instances. **c**, TRF weights from the full encoding model for a set of example neurons, demonstrating encoding of specific speech properties. Only feature class labels are shown (Extended Data Fig. 3 and Supplementary Table 1 show all individual feature labels). **d**, Unique variance for each class of speech feature on all significant neurons in each cortical site. Bar graphs show a breakdown of unique *R*^2^ for each neuron, which is derived from a comparison between variance explained by the full model and variance explained by a reduced model with a given feature class removed. Large pie charts show the proportion of explained variance attributed to each feature class across neurons. Small scatterplots (on the right) show the dominant feature for each neuron sorted by depth (the *x* axes are arbitrary for visualization). Coloured boxes around participant numbers indicate the dominant feature class for the site.

To quantify the tuning properties across all neurons, we used temporal receptive field (TRF) encoding models and variance partitioning^32^ (see ‘Encoding models’). TRF models predict neural activity from a combination of stimulus features at a set of lags, providing neuronal tuning curves that account for correlations among the stimulus features. We examined a broad set of speech features that we hypothesized could be encoded in STG: (1) acoustic–phonetic features^9^; (2) onsets from silence^31^; (3) intensity features including amplitude envelope and its derivatives (for example, the maximum rate of positive change in the envelope (peakRate)^11^); (4) speaker-normalized (relative) vocal pitch and its derivatives^10^; (5) lexical stress (correlated with intensity and pitch but coded here as a discrete label at the level of syllables)^33^; and (6) phoneme and word sequence probability^34,35^. Together, these features (Fig. 3a; Extended Data Fig. 3 and Supplementary Table 1 show the full feature set) allowed us to test the extent to which individual neurons and cortical sites encode different types of speech information.

STG neurons showed clear evoked responses to specific speech features. For example, some neurons responded to particular acoustic–phonetic features (such as vowels or voiced consonants (nasals)) (Fig. 3b; Extended Data Fig. 4 shows phoneme TRF weights for example acoustic–phonetic neurons), whereas others responded to acoustic cues, such as onsets from silence or peakRate events (Fig. 3b). Other neurons were responsive to high relative pitch, lexical stress or phoneme surprisal (Fig. 3b), illustrating that neurons in STG could be tuned to a large range of features included in this analysis.

To quantify encoding, we fit TRF encoding models with all 44 features (Extended Data Fig. 3 and Supplementary Table 1) for each neuron. We found many clear examples of neurons that were tuned to specific speech content, including particular groups of consonants or vowels, onsets from silence, low or high pitch, stress and sequence probabilities (Fig. 3c) (only feature class names are shown for visualization; each row corresponds to an individual feature within that class). Although some neurons exhibited significant weights for multiple classes of features (Methods discusses statistical quantification details), the overall pattern of encoding was sparse within each neuron.

All features together in these models explained variance up to *r* = 0.55 (mean = 0.182 ± 0.109; range = 0.0039–0.551). To understand how specific features contributed to this total explained variance, we characterized each neuron and each recording site according to the unique variance (*R*^2^) (ref. ^36^) for each of the six major classes of speech features (Fig. 3a) (acoustic–phonetic features were collapsed into vowels, voiced consonants and unvoiced consonants; only neurons with full model *r* value of greater than 95% shuffled permutation distribution are included) (‘Model comparisons’). We used unique *R*^2^ because it provides a robust and relatively conservative estimate of variance attributable to each group of features, which is critical for speech where many features are correlated with one another.

Each cortical site had one ‘dominant’ feature (determined by the largest slice in each main pie plot in Fig. 3d) that explained a significant proportion (25–62%) of the unique variance. Four sites were dominated by neurons encoding pitch (p1, p4-2, p6, p7; orange outline), whereas four were dominated by neurons encoding subgroups of acoustic–phonetic features (p2, p3, p5, p8; purple outline), and one site was dominated by neurons encoding onsets from silence (p4-1; light blue outline). These results demonstrate that different sites across the STG contain neuronal populations that are predominantly tuned to a particular speech feature across the vertical dimension, consistent with tuning observed at the cortical surface with ECoG^36^ (Extended Data Fig. 5). This finding was further corroborated by acoustic spectrogram decoding, which showed high-accuracy reconstructions and complementary information across recording sites (Extended Data Fig. 6 and Supplementary Text).

At the same time, we observed heterogeneity in encoding at every site. The dominant feature did not account for all explainable variance, and the remaining variance was split among the other classes of features (acoustic–phonetic, onset, intensity, relative pitch, stress and sequence probability).

We asked whether the heterogeneity in speech encoding patterns observed at each site could be explained by the encoding of different speech information in neurons at different cortical depths. For the four sites with strong relative pitch tuning (p1, p4-2, p6, p7), we found that relative pitch encoding was significantly stronger for superficial neurons, whereas acoustic–phonetic feature encoding was significantly stronger in mid-deep layers (Fig. 3d) (two-sample Kolmogorov–Smirnov test *P* < 0.05 in three of four sites). The patterns across depth at the other sites were less clear; however, these sites were more dominated by a single feature class (either acoustic–phonetic features or onsets). Thus, in some sites, the tendency for neurons tuned to features of the same class to colocalize appears to be one of the organizing principles across cortical depth in STG.

Overall, these results demonstrate that STG is organized according to sites with a dominant feature and that tuning within a site has a degree of heterogeneity that makes them not entirely modular^37,38^. This variation in speech feature tuning potentially facilitates local computations that integrate over the distinct aspects of speech that occur simultaneously^39^. Although each site exhibits dominant encoding of a particular feature, all sites contain a mixed population of neurons that encode different spectrotemporal information in contrast to largely homogeneous frequency tuning in cortical columns of the primary auditory cortex^40–42^.

## Speech responses across cortical depth

**Fig. 4:**
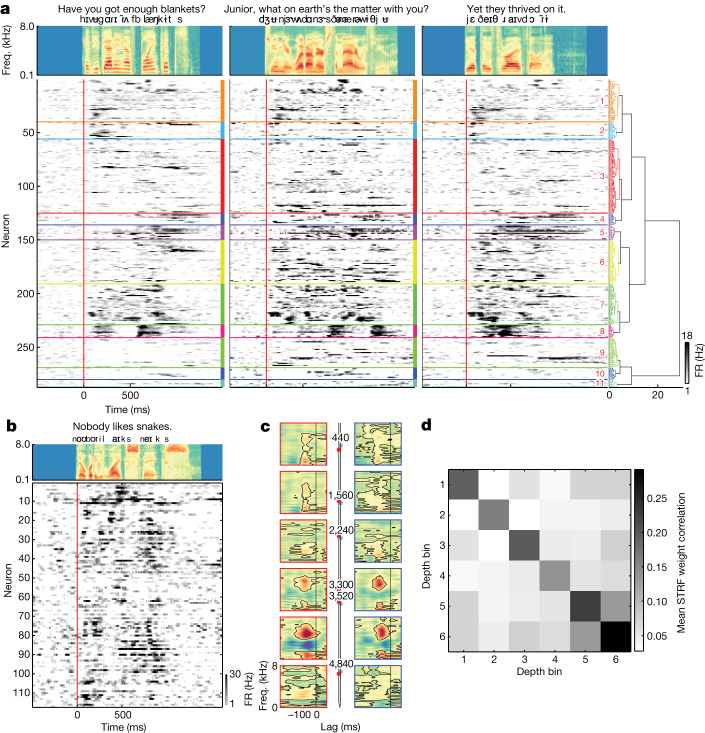
Neuronal activity is clustered by response type and cortical depth. **a**, Evoked responses for three example sentences for neurons with significant TRFs (Fig. 3) sorted by hierarchical clustering (Extended Data Fig. 7). **b**, An example PSTH from one site for one sentence (averaged over repetitions) shows variable response types at different depths. **c**, Example STRFs from one site show different tuning across depth and similar tuning for nearby neurons (left versus right). Numbers refer to neuron depth (micrometres). **d**, Correlation of STRF weights for neurons binned into six groups by depth (bin 1 is most superficial) averaged across all sites.

The heterogeneity we observed both within and across cortical sites demonstrates tuning to a highly diverse set of speech features in STG. To quantify the different types of responses that give rise to this tuning across the speech-selective neuronal population (*n* = 287; neurons with significant TRF encoding models), we examined neuronal activity using several complementary approaches. First, we compared the activity of each neuron with all other neurons (aggregated across recording sites) by computing the pairwise Pearson cross-correlations of the sentence-specific peristimulus time histograms (PSTHs) (Fig. 4a). Thirty percent of neuron pairs were significantly correlated (mean *r* = 0.28 ± 0.067, maximum *r* = 0.96, *n* = 287; *P* < 0.05, Bonferroni corrected).

We grouped neurons according to these correlations using hierarchical clustering and examined sentence-specific responses for all neurons and all clusters. This revealed several clusters in which most neurons were strongly correlated with one another (cluster 8 (100% of neuron pairs with significant correlations), cluster 7 (80%), cluster 5 (79.6%), cluster 1 (67.5%); all *P* < 0.05, Bonferroni corrected) (Extended Data Fig. 7). For clusters with highly correlated neurons, responses to individual sentences showed specific response dynamics, including onsets from silence (cluster 8) or broad sustained responses (cluster 5). Others showed increased firing rates throughout the sentences that were characterized by bursts of transient activity (cluster 7). These response types illustrate shared dynamics within subpopulations of neurons recorded from many different locations and depths along the STG, with sentence-specific responses embedded in these dynamics (Extended Data Fig. 8 and Supplementary Text have population state-space dynamics and speech feature decoding).

It is clear from single sentence-evoked activity that these response types and encoding patterns are organized as a function of depth (Figs. 1h and 4b). We directly examined columnar heterogeneity and the organization of neuronal representations across the cortical depth. We first asked whether correlations in average evoked responses were stronger for neurons that were anatomically closer to each other in depth. We found that in three of nine sites, there was a negative relationship between the peak cross-correlation (Supplementary Fig. 2) and the distance between neuron pairs (10 distance bins; p1: *r* = −0.098, *P* = 0.00095, *n* = 48; p3: *r* = −0.32, *P* = 1.5 × 10^−7^, *n* = 23; p7: *r* = −0.25, *P* = 9.1 × 10^−5^, *n* = 40).

Next, to quantify this relationship for both spiking activity and neural acoustic representations, we correlated the weights of individual neuron spectrotemporal receptive field (STRF) models^32^. In an example site, we observed that neurons at different depths exhibited very different tuning properties, with some neurons showing broadband spectral content (superficial), broad temporal responses (middle) and high spectral and temporal modulation (mid-deep) (Fig. 4c, left red). To understand whether neurons at similar depths were tuned similarly (that is, broader spatial organization across the cortical depth), we compared STRFs for different neurons that were at similar depths (Fig. 4c, right blue). Qualitatively, neurons at the same depth were very similar, whereas those farther away showed different tuning. We quantified this across all neurons by grouping each site into six depth bins and correlating the STRF weights. Averaged across all sites, we observed that neurons in the same bin were more similar compared with those in other bins (Fig. 4d; Extended Data Fig. 9 shows each individual site). We also found that neurons in mid-deep layers (approximately 3–4 mm) consistently had the shortest peak STRF latencies (less than 100 ms; analysis of variance *F*(4,190) = 3.8, *P* = 0.0058). Similar tuning for neurons at similar depths demonstrates a functional organization across cortical layers that complements the organization seen across the surface of STG, whereas differences in peak latencies could suggest that different layers receive distinct inputs^43,44^.

## Tuning of STG neurons to complex speech features

**Fig. 5:**
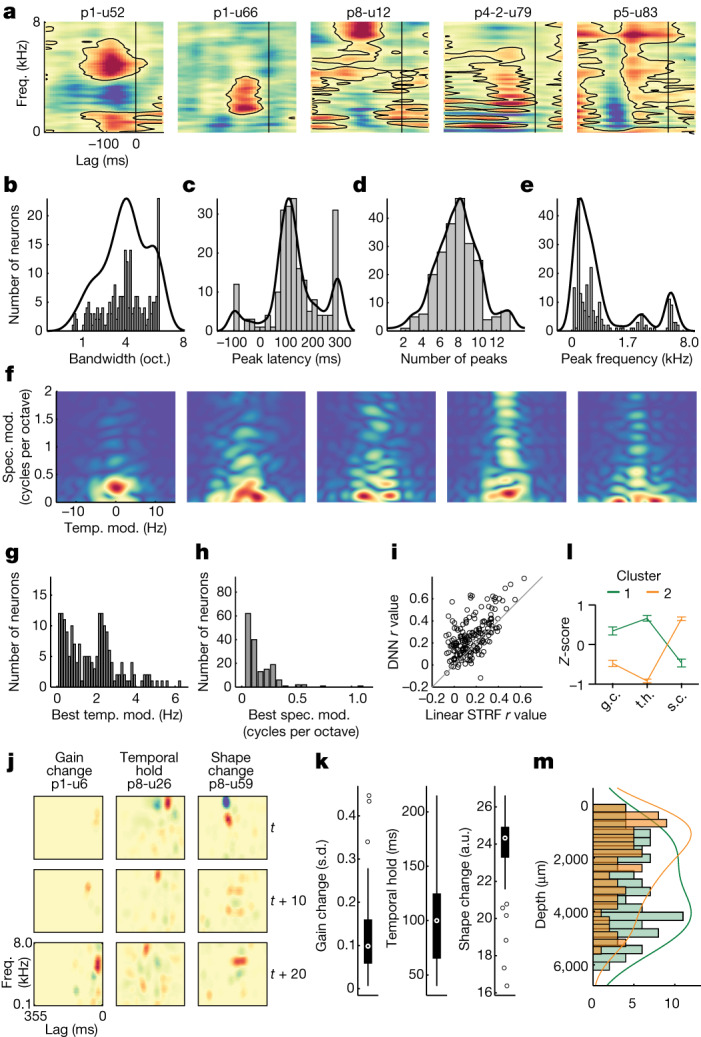
Encoding models reveal broad and diverse patterns of spectrotemporal tuning in STG neurons. **a**, STRFs for example neurons show distinct patterns of spectrotemporal tuning. **b**, Across all significant STRFs (permutation test versus shuffled distribution), tuning was broad, with mean bandwidth of approximately four octaves. **c**, STG neurons showed early-to-mid peak latency responses (approximately 150 ms). **d**, Most neurons had tuning to multiple spectral peaks. **e**, Frequency tuning was focused in the range of human voicing (less than 500 Hz). **f**, Modulation transfer functions for the same example neurons show diverse tuning for spectral and temporal modulations in speech. **g**, Across all neurons with significant STRFs, temporal modulations were focused at approximately 0.5 Hz and approximately 2.5 Hz. **h**, Spectral modulations were generally less than 0.5 cycles per octave. **i**, Comparison between linear STRF and DNN. **j**, Example dSTRFs for three neurons illustrate three types of nonlinearities: gain change, temporal hold and shape change. Rows are different time steps. **k**, Distribution of nonlinearities across the population of neurons with significant dSTRFs of each type (*n* = 189; box plots show the maximum and minimum values (whiskers), median (centre line) and the 25th to 75th percentiles (box limits)). **l**, Average (plus or minus s.e.m.) *Z*-scored nonlinearities for dSTRFs categorized using unsupervised hierarchical clustering (Supplementary Fig. 3) (cluster 1 *n* = 110, cluster 2 *n* = 79) showing high weight for one or two types of nonlinearities across the population. **m**, The two clusters have different distributions across cortical depth, with cluster 1 (gain change (g.c.)/temporal hold (t.h.)) being deeper than cluster 2 (shape change (s.c.)). oct., octave; spec. mod., spectral modulation; temp. mod., temporal modulation.

Spectrotemporal representations in STG at the level of ECoG are known to be complex and broad; however, it is unclear to what extent the same is true for individual neurons. Whereas the primary auditory cortex is associated with narrow-band frequency tuning^45^, we observed tuning profiles that were qualitatively far more complex in the STG (Fig. 5a and ‘Encoding model features’). For example, we found neurons with multipeak tuning at short lags (p1-u52), broad tuning to low- to mid-frequencies at short lags (p1-u66), increased firing to high-frequency content and decreased firing to low-frequency content at mid-latency lags (p8-u12), tuning to low-frequency harmonic structure at short to mid-lags (p4-2-u79) and broad spectral tuning with harmonic structure at mid-long lags (p5-u83). These spectrotemporal encoding patterns corresponded to acoustic–phonetic features, with individual neurons showing strong weights for groups of phonemes that share features (for example, high vowels, fricatives, plosives, nasals and so on) (Extended Data Fig. 4). These examples illustrate the range of spectrotemporal tuning of individual STG neurons, some of which were observed within a single site.

When we characterized all units with significant STRF models (permutation test versus shuffled distribution; *n* = 217, *r* = 0.039–0.45, mean = 0.17 ± 0.082) according to four key metrics of spectrotemporal tuning, we found (1) wide bandwidth (mean = 4.03 ± 1.57 octaves; Kolmogorov–Smirnov test versus uniform distribution: *D* = 0.92, *P* = 1.45 × 10^−38^) (Fig. 5b); (2) latencies characteristic of high-order auditory cortex (mean = 133 ± 99.9 ms; Kolmogorov–Smirnov test: *D* = 0.95, *P* = 3.09 × 10^−17^) (Fig. 5c); (3) multiple spectral peaks (mean = 7.72 ± 2.14; Kolmogorov–Smirnov test: *D* = 1.0, *P* = 5.86 × 10^−12^) (Fig. 5d); and (4) low-frequency tuning (median = 326.5 ± 1,989 Hz; KS test: *D* = 0.68, *P* = 2.92 × 10^−21^) (Fig. 5e). A bias towards lower frequencies may reflect the specialized nature of STG for human speech, where the majority of acoustic energy is in the voicing range (less than 500 Hz).

In addition to characteristic spectrotemporal patterns, speech sounds can also be described according to dynamic spectral and temporal modulation profiles, which are strongly correlated with speech intelligibility^46,47^. We computed the modulation transfer function (two-dimensional fast Fourier transform of the STRF^47^) and found that whereas some units showed primarily higher temporal modulation rates (approximately 2–4 Hz; for example, p1-u66) (Fig. 5f), others showed primarily higher spectral modulation rates (approximately 1–2 cycles per octave; for example, p8-u12 and p4-2-u79). Some neurons showed both high temporal and spectral modulation rates (for example, p5-u83). Across all neurons with significant STRF models, the rate of temporal modulation tuning was generally less than 4 Hz (mean = 1.64 ± 1.37; Kolmogorov–Smirnov test: *D* = 0.82, *P* = 1.01 × 10^−30^), with peaks at approximately 0.5 Hz and approximately 2.5 Hz (Fig. 5g). Spectral modulation tuning was generally less than 0.5 cycles per octave (mean = 0.15 ± 0.14; Kolmogorov–Smirnov test: *D* = 0.6, *P* = 2.05 × 10^−07^) (Fig. 5h). These temporal and spectral modulation rates are important for speech intelligibility, and this diversity of modulation tuning is similar to what is observed at the neural population level with ECoG^48^.

Although these results demonstrate robust encoding of spectrotemporal information across the population of neurons, STG neural populations are also characterized by non-linear representations^49^. To understand these nonlinearities in single neurons, we modelled the encoding functions with a deep neural network (DNN) (‘DNN model training’)^49,50^. Compared with the linear STRF, the DNN explained more variance in a majority of units, suggesting that many STG neurons have non-linear tuning (Fig. 5i).

Using the DNN, we extracted the dynamic spectrotemporal receptive field (dSTRF), which is the equivalent piecewise linear transformation, allowing us to interpret and visualize the model of a single neuron at a given instant as an STRF (‘dSTRF calculation and nonlinearity estimation’). Unlike an STRF, however, the dSTRF is context dependent, so its tuning changes for different time windows as the stimulus changes. Previous characterization of human ECoG in auditory cortex with this approach has identified three specific types of nonlinearities that improve model fits: (1) gain change (how much spectrotemporal tuning changes in magnitude in response to different input); (2) temporal hold (how much a model maintains the shape of its tuning while shifting it over lags in successive time steps); and (3) shape change (how much the shape of the spectrotemporal tuning changes after removing the effect of temporal hold)^49^. We found example neurons that were well explained by each of these nonlinearities (Fig. 5j). Across the population of neurons, each nonlinearity was characterized by a wide distribution, consistent with the heterogeneity we observed among these neurons (Fig. 5k).

Finally, to understand how these nonlinearities manifest across neurons, we performed unsupervised hierarchical clustering (minimum variance, Euclidean distance) on the three nonlinearities and identified two primary clusters of neurons (Supplementary Fig. 3). We found that one cluster primarily exhibited gain change and temporal hold, whereas the other primarily exhibited shape change (Fig. 5l). This also revealed that most neurons demonstrated high values for only one or two of the nonlinearities but not all three. In addition, neurons with high gain change and high temporal hold weights tended to be located in deeper cortical layers compared with neurons with high shape change weights (Fig. 5m) (*t*-test, *t*(188) = −3.88, *P* = 0.00014, *n* = 189; controlled for recording site). This suggests that the heterogeneity we observe across cortical layers (Figs. 3d and 4d) is at least partially a consequence of distinct non-linear computations relevant for speech encoding.

## ECoG activity reflects mixed neuronal contributions

High-frequency activity from direct neurophysiological recordings using surface ECoG has been critical to understanding human brain function across a variety of domains^51^, yet it is unclear to what extent signals recorded at the surface using ECoG macroelectrodes primarily reflect superficial neurons^15^ or whether there are also contributions from other neurons in deeper layers^16^.

**Fig. 6:**
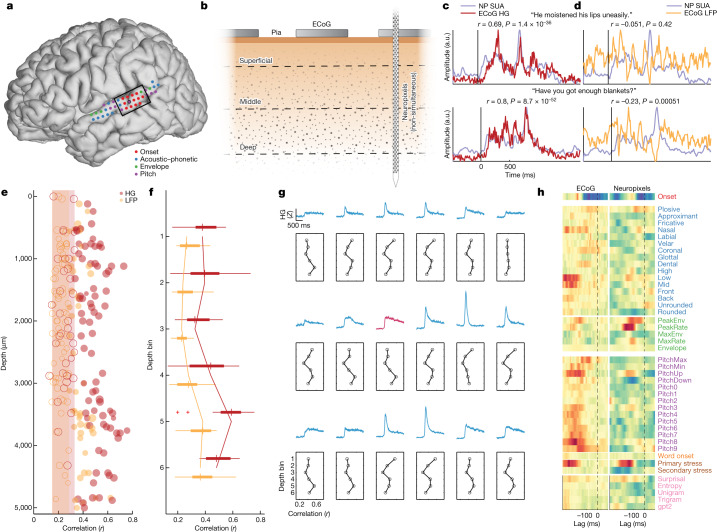
High-frequency population activity at the cortical surface reflects contributions from single neurons throughout the cortical depth. **a**, ECoG electrodes over STG from p1. Colour indicates the top feature in the speech encoding model for each ECoG electrode. The Neuropixels site is highlighted in black, and the black box notes the electrodes shown in **g**. **b**, Schematic of surface ECoG (macroelectrodes) and SUA across the cortical depth recorded with Neuropixels (not recorded simultaneously with ECoG). **c**, Example evoked responses to two sentences with average SUA from Neuropixels (NP) and ECoG high gamma at the same site in STG (Pearson *r*, two-sided test). **d**, Example evoked responses to the same two sentences with average SUA and ECoG LFP (Pearson *r*, two-sided test). **e**, Correlation between SUA PSTH activity and ECoG high gamma/LFP for each neuron in p1. Open circles and shaded regions indicate non-significance. **f**, Correlations in **d** (*n* = 117) binned into six depth ranges show contributions from all depths, particularly the deepest bins (box plots show the maximum and minimum values (whiskers), median (centre line) and the 25th to 75th percentiles (box limits)). **g**, Average evoked responses across sentences for ECoG electrodes across STG (top and middle traces; the red trace is the site of the Neuropixels probe). Bottom subplots show binned depth correlations as in **f**. **h**, TRF encoding model weights for ECoG (left) and average SUA (weighted by model *r*; right) show similar patterns. HG, high gamma.

We had the rare opportunity to record high-density surface ECoG (Fig. 6a) during inpatient epilepsy monitoring before intraoperative Neuropixels recording in some cases (Fig. 6b and ‘ECoG recording’). This allowed us to examine the relationship between neuronal signals across the cortical depth and population activity recorded from macroelectrode ECoG contacts on the pial surface. First, we compared the ECoG high-gamma responses to the same sentences with single-unit activity (SUA) averaged across all 117 units from p1 (PSTH calculated with a 10 ms window for comparison with high gamma) (Extended Data Fig. 10 shows an additional participant). We found a strong correlation, with both the overall shape of the response and individual peaks within the stimulus corresponding between the two signals (statistical details are in Fig. 6c). By contrast, the local field potential (LFP) signal from the ECoG arrays was less strongly correlated to SUA (Fig. 6d) (the statistical comparison between high gamma and LFP is quantified below), suggesting a specific relationship between high-frequency ECoG activity and neuronal spiking.

To address the relationship between surface ECoG signals and neurons at specific depths, we correlated activity between ECoG and SUA for each individual neuron and for both the high-gamma and LFP signals at the surface. First, we found that SUA was consistently more correlated with high gamma than with LFP^52^ (Fig. 6e). Second, we found significant correlations with high gamma throughout the depth (Fig. 6e) (mean *r* = 0.496 ± 0.102, range = 0.329–0.757; *n* = 82). We binned neurons into six equally spaced depth ranges and found that although there was a significant correlation to ECoG high gamma in all bins, deeper bins had stronger correlations (*F*(5,111) = 3.37, *P* = 0.0072) (Fig. 6f). This result contrasts with previous reports showing the strongest correlations between surface high gamma and superficial neuronal activity; however, these studies used only microelectrodes to record both signals^15^.

We also examined the correlation between depth-wise neuronal activity and ECoG high-gamma responses at electrodes throughout STG. Although it is unlikely that neurons at one site contribute measurable signals to ECoG electrodes several millimetres or centimetres away^53^, lateral connections and shared tuning properties may be related to organization seen at other sites. We observed strong correlations to most STG ECoG electrodes (*r* > 0.5), with the pattern across depth depending on the type of response. For example, the site directly over the Neuropixels probe exhibited heterogeneous tuning at the neuronal level (Figs. 3 and 4) and sustained average ECoG activity (Fig. 6g, red electrode), leading to the strongest correlations in mid-deep bins. By contrast, electrodes surrounding the Neuropixels recording site showed clear onset responses and correspondingly higher correlations to more superficial units (Fig. 6g).

Finally, we asked how the tuning of macroelectrode ECoG activity is related to the underlying neuronal population. We fit the same full speech feature encoding model (Fig. 3) on all STG ECoG electrodes and found organization for different speech features along the posterior–anterior axis of the gyrus (Fig. 6a). Consistent with previous work^36^, a zone in posterior STG was dominated by electrodes tuned to onsets from silence, whereas mid-anterior STG was characterized by acoustic–phonetic and prosodic features.

The site where we placed the Neuropixels probe showed a complex receptive field, with the strongest weights for onsets, acoustic–phonetic (low/mid vowels), envelope (acoustic edges), relative pitch (particularly low to mid pitch and rising pitch) and stress features (Fig. 6h, left panel). We compared this tuning profile with an average of models across neurons, weighted by the *r* value of the model (Fig. 6h, right panel). We observed a clear correspondence to the ECoG tuning, with strong weights on several key features, including onsets, envelope and stress (correlation between ECoG and Neuropixels models Spearman *ρ* = 0.166, *P* = 1.44 × 10^−12^). These results further support the claim that activity recorded at the pial surface with macroelectrodes reflects a complex mixture of the underlying neuronal population.

## Discussion

Here, we used large-scale single-neuron recordings enabled by the Neuropixels array to demonstrate the cellular encoding of speech processing in the human STG. Across the depth of cortex, the neuronal population is tuned to a dominant speech feature, consistent with the high-frequency broadband signal recorded at the surface with ECoG. At the same time, a relatively large proportion of neurons throughout the vertical cortical column also encode a large variety of other speech features, revealing a distinct, previously unappreciated dimension for speech encoding.

Our observations in STG contrast with ‘columnar’ recordings in the primary auditory cortex, where neurons across the cortical layers exhibit tuning to the same narrow-band frequency^40,41^. STG neurons instead encode a wide variety of complex spectrotemporal, phonetic and prosodic features^5^, and they tend to exhibit correlated tuning at locally adjacent depths. The dense sampling across depth provided by Neuropixels probes enables investigation of these fundamental organizational questions^38^.

Our results contribute to an emerging model of the three-dimensional functional organization of the human STG. Specifically, mid-deep cortical layers, which are most strongly correlated with the surface ECoG response^16^, show the fastest responses for a given site, possibly reflecting direct thalamic inputs^54,55^. Across cortical layers, local clusters of neurons are tuned to specific classes of speech information (for example, acoustic–phonetic or prosodic), possibly reflecting lateral inputs from other sites^42,56–61^. The unique functional organization of associative auditory areas such as STG, where a dominant feature is encoded alongside other speech features, could have an important role for local computations and integration of complex signals, such as those in spoken language.

The application of Neuropixels has the potential to be transformative for the next generation of human neuroscience. The present demonstration of large-scale neuronal recordings will greatly accelerate our understanding of the unique computations and representations of the human cortex.

## Methods

### Participants

Participants (three female, five male; ages 33, 28, 24, 42, 53, 67, 22 and 34 years) underwent clinical surgery for resection of epilepsy focus or brain tumour (Supplementary Table 2). Before surgery, participants were consented for temporary intraoperative placement of Neuropixels probes during the procedure. In all cases, the tissue where Neuropixels probes were inserted was resected according to the surgical plan. The cortical locations were evaluated with electrical stimulation mapping and determined to not be critical for language. In one case, a positive stimulation mapping site was resected because of severe seizures.

### Participant consent

All protocols were reviewed and approved by the University of California, San Francisco Institutional Review Board. Patients gave informed consent before surgery for temporary intraoperative placement of Neuropixels probes during the procedure.

### Neuropixels hardware and probe placement

Neuropixels 1.0 NHP-short probes with 10 mm long shanks and metal dovetail caps (IMEC) were used for all recordings. Two 27 gauge subdermal needle electrodes (Ambu) were soldered separately to the probe flex interconnect to serve as ground and reference using lead-free solder and two strands of twisted 36AWG copper wire. Details of the hardware configuration have been previously reported in ref. ^2^.

Electrode placement was determined after clinical mapping and the resection zone had been defined. In each case, probes were inserted into tissue that had been targeted for resection and was subsequently removed during the same surgery.

We used methods described in our previous work^2^ to position and advance the probe into tissue. In brief, once a site had been identified, the probe was positioned perpendicularly to the cortical surface and advanced slowly with a micropositioner until we reached a depth of approximately 7 mm. This distance was chosen to allow us to cover the full depth of the cortex with the ‘long column’ electrode montage on the Neuropixels probe. We attempted to leave approximately 600–700 μm of active recording channels outside of the brain so that we could identify the cortical surface on the recordings, which we used to estimate the depth of the recorded units. Cortical pulsations related to cardiac and respiratory cycles were dampened using surgical patties or a pedestal attached to the micropositioner. Post hoc motion correction was applied using Kilosort 2.5 (ref. ^19^), and stability of units was verified manually during the sorting process.

Recordings were typically limited to 10–15 min. All participants were awake during recording.

### Preprocessing and spike sorting

Automated spike sorting was performed on the high-pass filtered data (cutoff 300 Hz, sampling rate 30 kHz) with Kilosort 2.5 using standard parameters^19^. The output of Kilosort was then manually curated by at least two researchers using Phy. Clusters with abnormal spike waveforms, excessive interspike-interval violations or multiple waveform shapes that could not be split into separate clusters were rejected. The remaining clusters were labelled as putative single units and were included in subsequent analyses. Spike waveforms were subsequently clustered into putative cell types (Fig. 1g), which were well separated into three clusters (Supplementary Fig. 4). PSTHs for data visualization and encoding models were calculated using a 50 ms sliding window, except where noted. Although we planned to use the LFP signal to identify layer boundaries using current source density analysis^14^, technical issues precluded obtaining clear LFP signals; therefore, we focus here on single-neuron spiking activity.

### Speech stimuli and procedures

One hundred and ten unique sentences from the TIMIT corpus^18^ were played to participants during surgery. Ten of these sentences were repeated 10 times each, providing 200 total trials. Sentences had a mean duration of 1.72 s (s.d. = 0.394 s) and were produced by 103 unique male and female speakers. In two participants (p2 and p3), the full set of stimuli was repeated once, providing 400 trials (and therefore, allowing up to 20 repeats of certain sentences) (Fig. 2).

### ECoG recording

Before the resection surgery, a subset of participants received inpatient care in the University of California, San Francisco Epilepsy Monitoring Unit, where activity was recorded using high-density (4 mm pitch) subdural ECoG electrode arrays. Four participants listened to the same sentence stimuli used in the intraoperative Neuropixels experiments while ECoG was recorded. The location of the intraoperative Neuropixels insertion was carefully matched to the corresponding sites of the ECoG electrodes using surface vessel and sulcal landmarks. ECoG recordings were referenced online to a subgaleal reference electrode and were not re-referenced for analysis.

Following previously published methods, we extracted ECoG activity in the high-gamma (70–150 Hz) frequency range using Morlet Wavelets. We also examined broadband activity from the LFP signal, applying minimal filtering (notch filters at 60 Hz and harmonics up to 500 Hz).

### Tissue resection and immunohistochemical staining

The cortical tissue surrounding the Neuropixels insertion site was surgically removed. When possible, the tissue was resected en bloc in a single piece for histological analysis (Extended Data Fig. 1). In some cases, the STG was too narrow or had arteries that needed to be preserved, precluding en bloc excision.

### Encoding model features

To quantify single-neuron speech encoding, we used multiple complementary descriptions of the sentence stimuli, which allowed us to examine spectrotemporal-, phonemic-, acoustic–phonetic-, prosodic- and sequence-level features.

To examine spectrotemporal features, each sentence was decomposed into 80 frequency bands, which were logarithmically spaced using the mel scale to match the perceptual characteristics of the peripheral auditory system.

To examine encoding of acoustic–phonetic-, prosodic- and sequence-level features, we annotated each sentence using a combination of hand-labelled and automatically transcribed features. In total, we annotated each speech stimulus with 44 features, which were organized into six major categories: (1) sentence onsets^31^; (2) acoustic–phonetic features^9^; (3) relative pitch^10^; (4) amplitude envelope^11^; (5) stress^33^; and (6) speech sequence statistics^34,35^. Extended Data Fig. 3 shows example annotated sentences, and Supplementary Table 1 shows descriptions of each feature.

Sentence onsets were coded as binary variables at the first sample of each sentence. Acoustic–phonetic features reflect the manner and place of articulation of each speech sound, and they were coded as binary variables for manner of articulation (plosive, approximant, fricative and nasal); place of articulation for consonants (labial, velar, coronal, glottal and dental); and vowel features (high, mid, low, front, back, unrounded and rounded).

**Extended Data Fig. 2:**
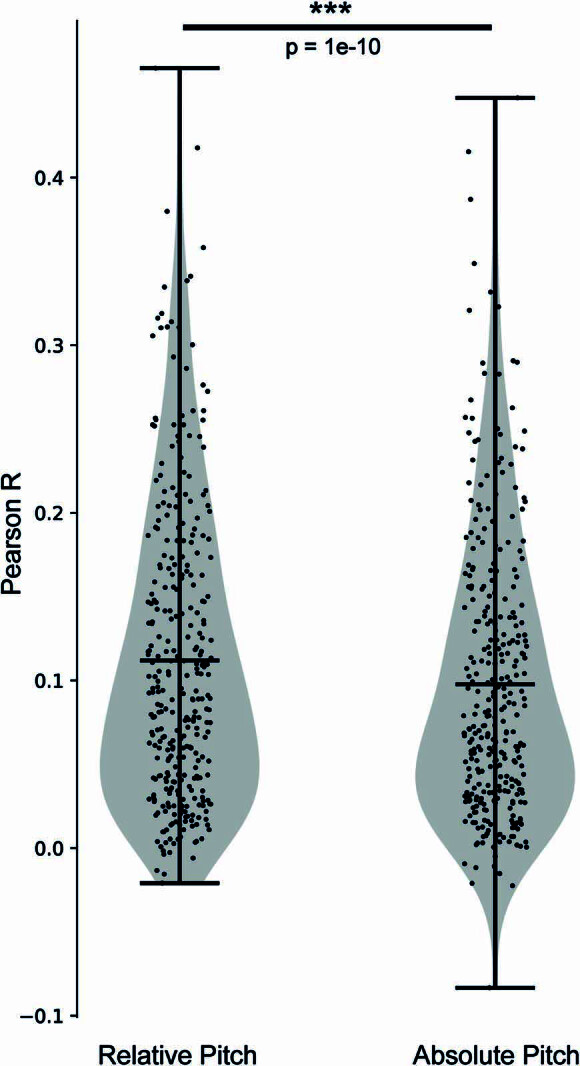
Relative versus absolute pitch encoding. The reconstruction accuracy of each neuron (y-axis, Pearson r-value; mean±range of violin plots) is plotted for a model that uses just relative pitch to predict neural activity in each neuron (left), or just absolute pitch (right). As expected, the two predictions are highly correlated (Pearson r = 0.89; p = 5^−116^), given that relative and absolute pitch are highly correlated in the stimulus. Despite the high correlation, relative pitch explains neural activity significantly better than absolute pitch (paired samples two-sided t-test, t = 6.5; p = 1^−10^; *n* = 322). This is in line with ECoG studies, which show that STG encodes relative pitch to a greater extent than absolute pitch (e.g., ^10^), whereas primary auditory cortex is more dominated by absolute pitch^36^. The combined precedence of relative pitch encoding in STG, and the dominance of relative pitch over absolute pitch in our targeted analyses, motivates our choice to focus on relative pitch in this work.

**Extended Data Fig. 3:**
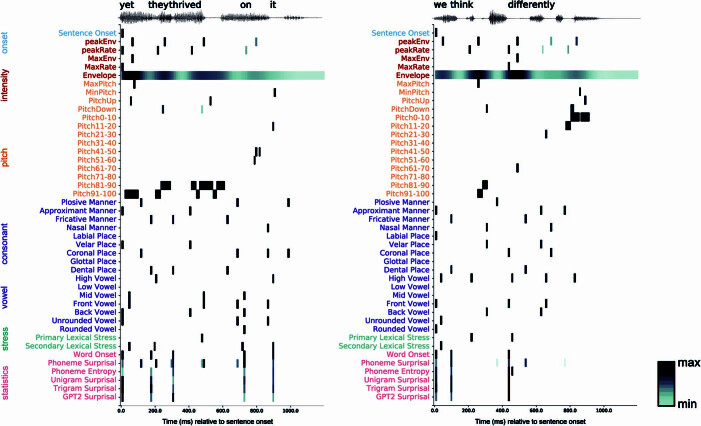
Stimulus annotation examples. Full feature annotation for two sentences. X-axis corresponds to time relative to sentence onset. Y-axis corresponds to each of the 44 features in the encoding model. Colour of the y-axis labels indicates the feature class.

**Extended Data Fig. 4:**
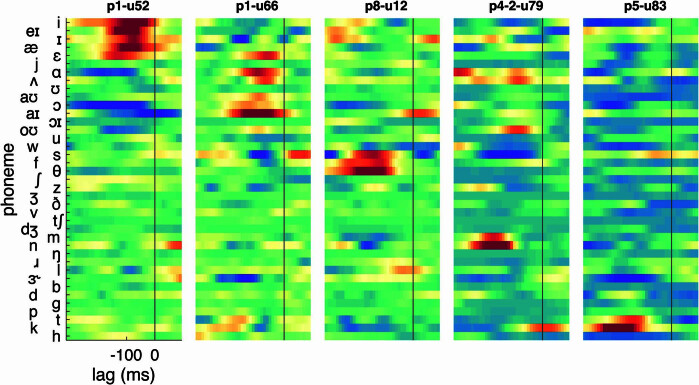
Phoneme TRF encoding weights for example neurons in Fig. 5. For the example neurons in Fig. 5, we fit a TRF encoding model with 39 phonemes as features. We found that different spectro-temporal and modulation patterns corresponded to different groups of phonemes. For example, we observed neurons that were tuned to specific vowels like /i/, /eɪ/, /ɪ/, /æ/, and /ɛ/ (p1-u52), which are mid-high vowels characterized by relatively low F1 and high F2 formants (Fig. 5a). In contrast, other units were tuned to a different set of vowels including /ʌ/, /ɑ/, /ɔ/, and /aɪ/ (p1-u66), which are mid-low vowels with relatively high F1 and low F2 formants (Fig. 5a). Other neurons were tuned to different groups of consonants like /s/, /f/, and /θ/ (p8-u12), which are fricatives characterized by high frequency content (Fig. 5a). Others were tuned to consonants like /m/ and /n/ (p4-2-u79), which are nasal sounds. Finally, some neurons were tuned to consonants like /t/ and /k/ (p5-u83), which are plosive sounds characterized by high temporal modulations. These examples (see also Fig. 2) illustrate that single STG neurons encode acoustic-phonetic features, rather than individual phonemes^69,70^.

**Extended Data Fig. 5:**
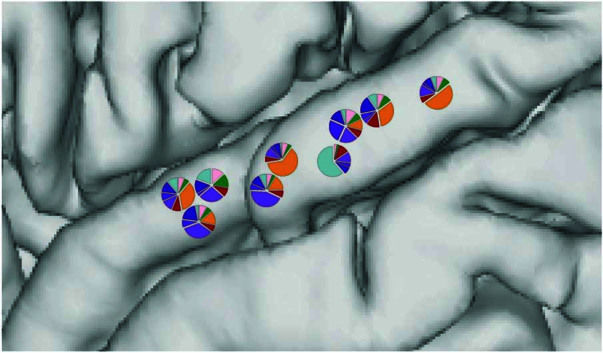
Site-specific tuning across the surface of STG. Pie plots are reproduced from Fig. 3d, plotted on the approximate location of each recording site from Fig. 1b. Locations have been shifted slightly to maximize visibility of each pie plot.

**Extended Data Fig. 6:**
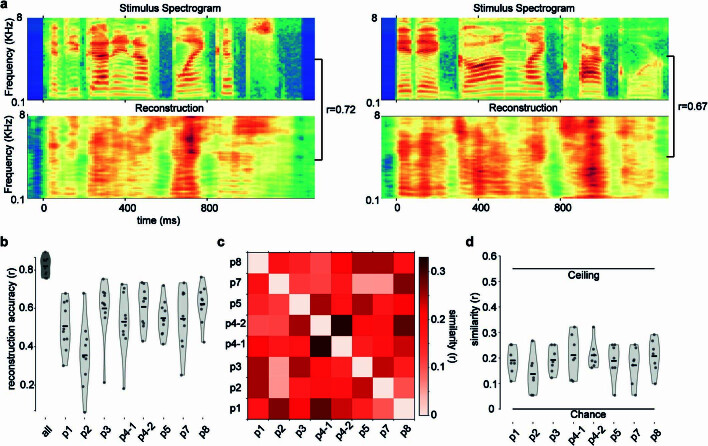
Stimulus reconstruction from population activity. **a**: Stimulus spectrograms for two sentences (top), reconstructed using a linear model with 290 principal components (bottom), derived from 623 neurons (p6 was excluded due to having less data). Correlations between original and reconstructed spectrograms are relatively high (r ~ 0.7). **b**: Stimulus reconstruction accuracy (Pearson r-value) for each of the ten repeated sentences (individual dots). Accuracy is highest when using neurons from all sites (dark bar), and lower but still relatively strong for each individual site separately. Small black dashed line in the violin plots represents the mean performance from each population across sentences. **c**: Pairwise similarity (Pearson r-value) of stimulus reconstructions across individual sites. Sites recorded from the same participant (p4) are the most similar. **d**: Similarity (Pearson r-value) of predictions across sites, as compared to ceiling and chance performance when using all 623 neurons from all sites. Dots are the other recording sites correlated with the site indicated on the x-axis. In all cases, mean similarity is between chance and ceiling, indicating that all sites reconstruct some, but not all, similar spectrotemporal information.

**Extended Data Fig. 7:**
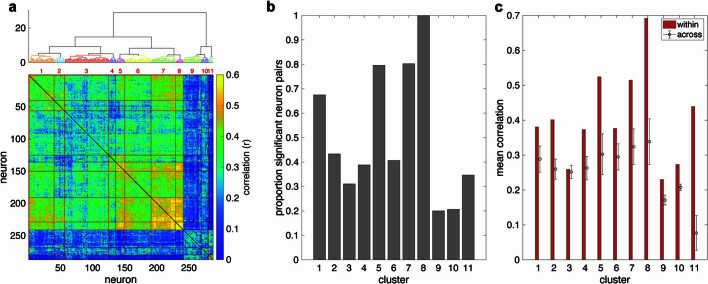
Hierarchical clustering of neuronal response correlations. **a**: Pairwise peak cross-correlation among neurons from nine recording sites shows groups of highly correlated response dynamics. Matrix sorted by hierarchical clustering (top). **b**: Proportion of neurons in each cluster that are significantly (p < 0.05, two-sided test, Bonferroni corrected) correlated with other neurons in the cluster. **c**: Within-cluster (red) and across-cluster (black; mean±s.e.m.; *n* = 11 clusters, 287 neurons) correlations.

**Extended Data Fig. 8:**
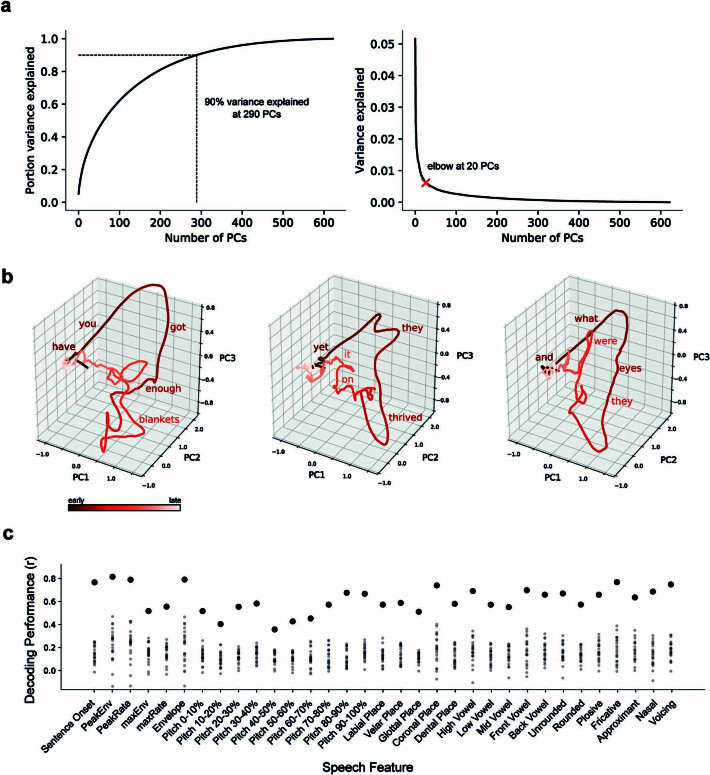
Population state-space dynamics and speech feature decoding. **a**. Principal component analysis (PCA) performed on 623 single neurons (data from one participant was excluded due to fewer sentences). 90% of the total variance was explained with 290 PCs (46.5% of the full dimensionality of the data). Additionally, an elbow in variance was found at approximately 20 PCs, demonstrating the relatively low dimensionality of the population data. **b**. Population state-space visualizations for three example sentences. The first three PCs are plotted with the time course of each sentence (averaged over 10 repetitions). Colour from dark to light reflects time relative to sentence onset. All sentences show highly similar trajectories (PC1 Pearson r-value across 10 sentences mean=0.78 ± 0.17; PC2 mean=0.88 ± 0.08; PC3 mean=0.65 ± 0.24). **c**. Speech feature decoding performed on acoustic-phonetic, intensity, and relative pitch features. All features are significantly decodable above chance (small dots are shuffled models, large dots are the true model for each feature).

**Extended Data Fig. 9:**
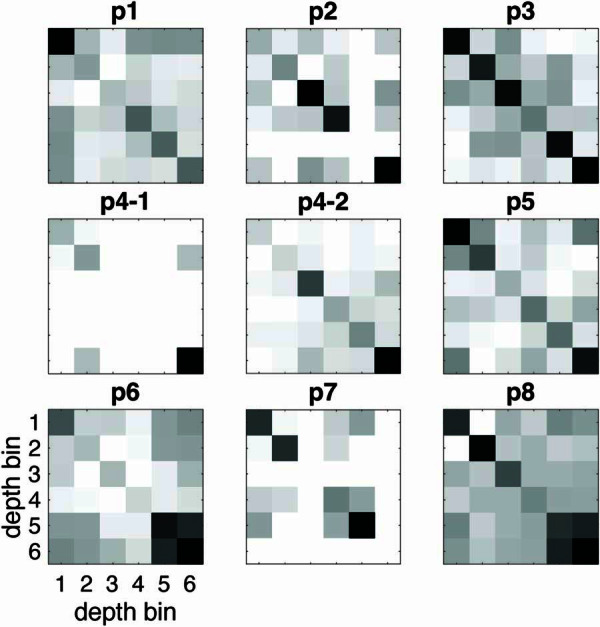
Encoding model similarity by depth for each individual site. Correlation of STRF weights for neurons binned into six groups by depth. In some sites, we did not sample neurons in every depth bin (white).

**Extended Data Fig. 10:**
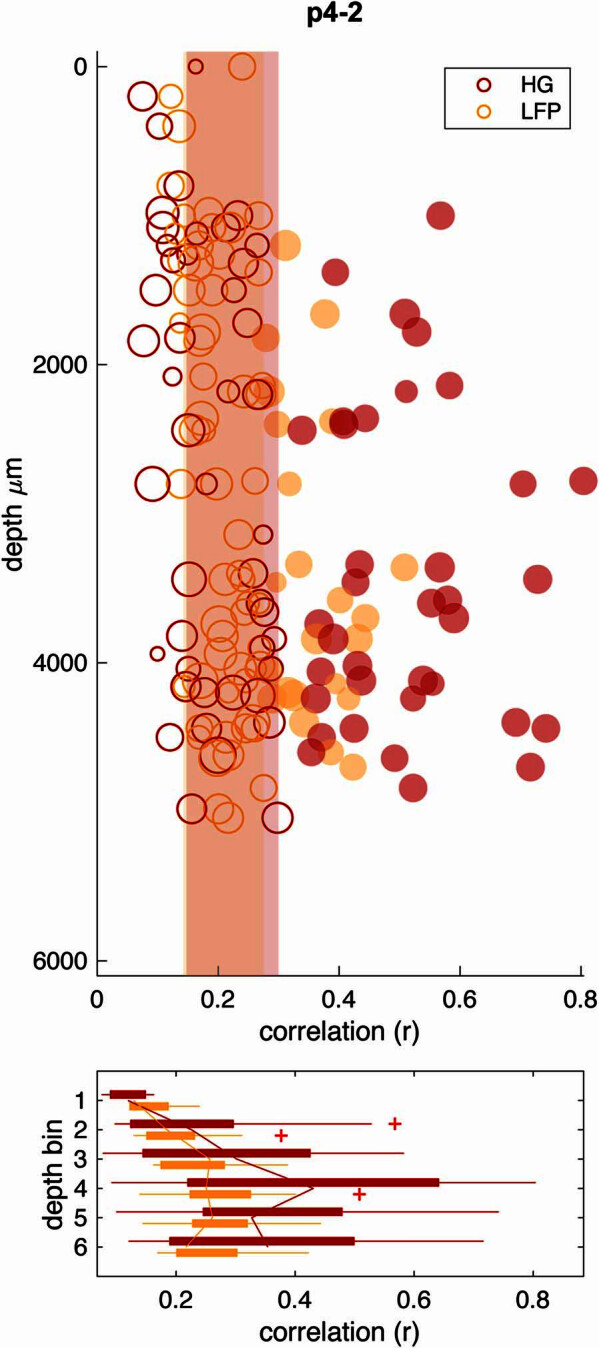
Correlation between surface ECoG and SUA in p4-2. Correlation between SUA PSTH activity and ECoG HG/LFP for each neuron in p4-2 (*n* = 82). Open circles/shaded regions indicate non-significance. Bottom: Correlations binned into six depth ranges show strong contributions from all depths, particularly the mid-deep bins (boxplots show the maximum and minimum values [whisker], median [centre line] and the 25th to 75th percentiles [box limits]).

For relative vocal pitch, we focused on speaker-normalized pitch, which has been shown to be encoded in STG using ECoG^10^. In preliminary analyses, we also considered absolute pitch (in hertz); however, we found that the vast majority of neurons were better explained by relative pitch. When we tested a model fit on just relative pitch or just absolute pitch, relative pitch better explained neuronal responses (paired samples *t*-test *t* = 6.5, *P* = 1 × 10^−10^) (Extended Data Fig. 2). To reduce redundancy in the models, we therefore excluded absolute pitch. To compute pitch, we used the ‘crepe’ Python module^62^ (v.0.0.12). The resulting pitch values were normalized within speakers between zero and one, representing the minimum and maximum of that speaker’s own pitch range. These normalized relative pitch contours were quantized into 10 equally-sized bins. In addition, we also included derivative measures of pitch (increasing/decreasing and maximum/minimum within each sentence), which describe how the pitch contour changes. The relative pitch feature is continuous over time, with a sample rate of 100 Hz.

The amplitude envelope of speech was characterized using five features: (1) the continuous speech envelope defined as the rectified Hilbert transform of the speech waveform with a low-pass 10 Hz Butterworth filter; (2) binary impulses at the peaks in the envelope; (3) a single impulse at the maximum peak of the envelope for each sentence; (4) peaks in the positive derivative of the envelope (peakRate^11^); and (5) the maximum derivative point for each sentence.

Although relative pitch and amplitude are characteristic acoustic properties of stress in speech (in addition to duration, which was coded implicitly in the acoustic–phonetic features), we also marked the moments of primary and secondary syllabic stress^33^ according to manual annotations from the TIMIT corpus^18^ using binary impulses.

Finally, we included two types of speech statistics. At the phoneme level, we coded surprisal and entropy of each speech sound as a function of the previous speech sounds in the word^63^. Impulses were placed at phoneme onset, matching the temporal coding of phonetic features. These metrics were computed based on word frequency counts from the English Lexicon Project^64^. At the word level, we included Unigram surprisal (word frequency), trigram surprisal (likelihood of a word given the two previous words) and surprisal from a larger contextual window (computed from GPT2, a large language model that accounts for long-distance dependencies in language^65^). These word-level statistical features were coded with impulses at word onset. To account for the effect of word-level statistics being defined only at word onsets, we also coded binary impulses at word onsets.

### Encoding models

For all types of stimulus description, we modelled single-neuron responses using a ridge regression TRF^36^ with L2 regularization. Neural responses were characterized using the PSTH with a 50 ms smoothing kernel.

Linear receptive field models were fit using the following framework:

$$\hat{x}(t)={x}_{0}+\sum _{f}\mathop{\sum }\limits_{\tau =0}^{T}\beta (\tau \,,f)S(\,f\,,t-\tau ),$$

where *x* is the PSTH of each neuron, $\beta (\tau \,,f)$ is the regression weights for each feature *f* at each time lag *τ* and *S* is the stimulus representation for feature *f* at time *t* − *τ*. We used a time window that ranged from 300 ms before to 100 ms after.

Each model was fit using ridge regression:

$$\widehat{\beta }={\rm{argmin}}{{\rm{| | }}\,y-XB{\rm{| | }}}_{2}^{2}+\lambda {{\rm{| | }}B{\rm{| | }}}_{2}^{2},$$

where *β* *∈* *R*, *X* is the set of stimulus features, *B* is the set of regression weights learned in the equation above and *λ* is the L2 penalty term used to minimize the values of each *β*. *λ* was evaluated over a range from 1 × 10^3^ to 1 × 10^9^ with logarithmic spacing and was chosen to maximize model performance on held-out data while also yielding temporal smoothness similar to the underlying data.

All stimulus features were normalized by first transforming their range between zero and one and then to control for the relative sparsity of different features, by dividing their amplitude by their mean over time:

$${\rm{bounded}}=\frac{s-\min (s)}{\max (s)-\min (s)}$$

$${\rm{normalized}}=\frac{{\rm{bounded}}}{{\rm{mean}}({\rm{bounded}})}.$$

Models were fit by concatenating sentences, allowing 200 ms of silence between trials. The TRF model was trained on 80% of the data and evaluated on 20% of the data using a Pearson correlation between the predicted and true PSTHs for each unit. We repeated this procedure 50 times on different random shuffles of the data.

To evaluate statistical significance, we compared the true model fit with a null model fit on temporally permuted data. For the null model, we shuffled the within-sentence neural data and speech features by a random lag between −500 and 500 ms. This retained the covariance structure between the features in the model and matched general distributional properties of the data to form a fair and conservative comparison with the true model fits. We repeated this 50 times, with random shuffles of the trials. Each null model was also fit using ridge regression as above.

A neuron was considered to have significant encoding if the average performance of the true model over 50 repetitions exceeded at least 47 of the repetitions (*P* < 0.05) of the temporally shuffled random models.

### Model comparisons

To quantify the unique contribution of each class of feature in the ‘full’ model (sentence onsets, acoustic–phonetic features, relative pitch, amplitude envelope, stress and sequence statistics), we compared the model with all 44 predictors to a reduced model where the predictors of a given class of features were removed. For example,

$${r}_{{\rm{u}}{\rm{n}}{\rm{i}}{\rm{q}}{\rm{u}}{\rm{e}}({\rm{o}}{\rm{n}}{\rm{s}}{\rm{e}}{\rm{t}})}={r}_{{\rm{o}}{\rm{n}}{\rm{s}}{\rm{e}}{\rm{t}}+{\rm{a}}{\rm{c}}{\rm{o}}{\rm{u}}{\rm{s}}{\rm{t}}{\rm{i}}{\rm{c}}-{\rm{p}}{\rm{h}}{\rm{o}}{\rm{n}}{\rm{e}}{\rm{t}}{\rm{i}}{\rm{c}}+{\rm{p}}{\rm{i}}{\rm{t}}{\rm{c}}{\rm{h}}+{\rm{i}}{\rm{n}}{\rm{t}}{\rm{e}}{\rm{n}}{\rm{s}}{\rm{i}}{\rm{t}}{\rm{y}}+{\rm{s}}{\rm{t}}{\rm{r}}{\rm{e}}{\rm{s}}{\rm{s}}+{\rm{s}}{\rm{e}}{\rm{q}}{\rm{u}}{\rm{e}}{\rm{n}}{\rm{c}}{\rm{e}}{\rm{s}}}-{r}_{{\rm{a}}{\rm{c}}{\rm{o}}{\rm{u}}{\rm{s}}{\rm{t}}{\rm{i}}{\rm{c}}-{\rm{p}}{\rm{h}}{\rm{o}}{\rm{n}}{\rm{e}}{\rm{t}}{\rm{i}}{\rm{c}}+{\rm{p}}{\rm{i}}{\rm{t}}{\rm{c}}{\rm{h}}+{\rm{i}}{\rm{n}}{\rm{t}}{\rm{e}}{\rm{n}}{\rm{s}}{\rm{i}}{\rm{t}}{\rm{y}}+{\rm{s}}{\rm{t}}{\rm{r}}{\rm{e}}{\rm{s}}{\rm{s}}+{\rm{s}}{\rm{e}}{\rm{q}}{\rm{u}}{\rm{e}}{\rm{n}}{\rm{c}}{\rm{e}}{\rm{s}}}$$

$$\begin{array}{l}{r}_{{\rm{unique}}({\rm{acoustic}}-{\rm{phonetic}})}={r}_{{\rm{onset}}+{\rm{acoustic}}-{\rm{phonetic}}+{\rm{pitch}}+{\rm{intensity}}+{\rm{stress}}+{\rm{sequences}}}\\ \,-{r}_{{\rm{onset}}+{\rm{pitch}}+{\rm{intensity}}+{\rm{stress}}+{\rm{sequences}}}\end{array}$$

and similarly for the other groups of features.

We report units as having significant unique *R*^2^ when both the unit had significantly better model fits than the temporally shuffled data and the unique *R*^2^ over repetitions was significantly higher than the shuffled data at *P* < 0.05 using a rank sum test.

### DNN model training

The DNN model was a five-layer convolutional neural network with 512 kernels of sizes 5, 5, 7, 9 and 11; dilations of one, one, one, two and four; rectified linear unit (ReLU) activations; and a linear projection layer to predict all neuron responses (PSTH with an 80 ms window) simultaneously. Only the projection layer had a bias term. This model, therefore, had a receptive field of 71 lags or 355 ms. The objective function during training was the mean squared error between predicted and true responses averaged across units, and we used the Adam optimizer with weight decay of 0.003, an initial learning rate of 0.0001 and an exponential learning rate decay of 0.996. As a more fair comparison with the DNN, we trained linear models using the same data and gradient descent optimization (Fig. 5i).

Both DNN and linear models were trained with a jackknifing procedure, where 10 models were trained by leaving out 10% of the training data for each. When evaluating the models, the predictions of all 10 models on the test stimuli were averaged to produce a single response for each neuron.

### dSTRF calculation and nonlinearity estimation

For a convolutional neural network with ReLU activations and no intermediate bias terms, the dSTRF can be computed as the gradient of the output with respect to the input vector^49,50,66^. We used automatic differentiation in PyTorch^67^ to compute this gradient for each of the jackknifed models on the test stimuli. To ensure robustness of the dSTRFs, the 10 dSTRFs were averaged and further filtered based on sign consistency, whereby all 10 models were required to agree on the sign of a given lag-frequency bin in the dSTRF for a given input; otherwise, the averaged bin was set to zero.

To estimate gain change for each neuron, the Frobenius norm of the dSTRF was calculated at each time point, and the gain change was defined as the norm’s s.d. over the duration of the stimulus, providing an estimate of how much the magnitude of the dSTRF changes over time.

The temporal hold nonlinearity of a dSTRF describes the largest duration in time that a spectrotemporal pattern persists in the dSTRF through shifts in lag over successive time steps. Estimating the temporal hold of a given dSTRF requires multiple steps. For the lag-frequency dSTRF at time *t* (dSTRF_*t*_(*τ*, *f*)) for each lag *n*, up to the maximum lag size of the dSTRF, we computed its correlation with the future dSTRF (dSTRF_*t*+*n*_(*τ*, *f*)) and its correlation with the shift-corrected dSTRF (dSTRF_*t*+*n*_(*τ* − *n*, *f*)). For each *n*, a one-tailed Wilcoxon signed-rank test was used to determine if there was a significant positive change between the latter and the former correlations across all time *t*. The temporal hold was defined as the largest lag *n* yielding a significant test statistic.

The shape change nonlinearity describes the heterogeneity of the spectrotemporal tuning functions used by the DNN model beyond gain change and temporal hold. First, the dSTRF for a given neuron was shift corrected by lag aligning the dSTRF over time to the average dSTRF over the stimulus. To do this, we used an iterative approach. On a single iteration, for each time *t*, we found the best shift *nt*, which maximized the correlation between the shifted dSTRF (dSTRF_*t*_(*τ* − *nt*, *f*)) and the average dSTRF. At the end of the iteration, the new average dSTRF was computed after shifting each dSTRF_*t*_ by its best shift. Iterations continued until either the best shifts converged or a maximum of 100 iterations were performed. Then, with these shift-corrected dSTRFs, we computed the complexity of the dSTRFs over time. This complexity was estimated using the sum of the singular values of the dSTRFs, normalized by their maximum. Because singular values specify the variance of each corresponding vector, dSTRFs whose sorted singular values decay more slowly and therefore, have a higher sum after normalization encompass a broader set of spectrotemporal tuning functions.

Before performing clustering, nonlinearities were *Z*-scored, and outliers were compressed toward the mean through the transformation tanh(*x*/2.5) × 2.5 to give them comparable magnitudes. The code for estimating dSTRFs and nonlinearities can be found on GitHub^49^.

### Population state space and dynamics

We applied principal component analysis to the single-neuron activity of all participants who listened to the full 10 repetitions of 10 sentences (8 subjects, 623 neurons). We fit principal components on the PSTH of the concatenated repeated sentences. We visually determined the ‘elbow’ by plotting the ranked explained variance across all components. We averaged the PSTH across repetitions of the same sentence and projected this onto the principal component manifold for each different sentence. Similarity between each principal component was quantified by computing the Pearson correlation between the principal component time course of a given sentence average.

### Stimulus spectrogram reconstruction

We fit TRF ridge regression models on the 290 principal components that explained 90% of the variance in the PSTH. The model output was the 80 mel frequency bands of the speech spectrogram over time. We used an alpha regularization parameter of 500 using the ‘ReceptiveField’ function implemented in mne-python v.0.22.0. Time delays of −300 to 100 ms were used, with a sample frequency of 100 Hz. We fit the model on responses to nine distinct sentences and evaluated on one held-out sentence. Performance was quantified using Pearson correlation between the predicted time course and the true time course for each frequency and then averaged across the 80 frequency bands^68^.

To compute the reconstruction accuracy on a site-by-site basis, we fit the TRF models on all neurons from a given site separately using a leave-one-sentence-out cross-validation scheme. We compared this with the performance of a TRF model fit on all 623 neurons.

To compare the similarity between the stimulus reconstructions obtained from each site, we concatenated responses to all sentences together and correlated the spectrotemporal time course predictions. Ceiling was computed as the reconstruction accuracy of the ground truth when concatenating all sentences together and fitting a model on all 623 units. Chance was computed by shuffling the sentence order and comparing the ground truth concatenated sentences with the predicted (shuffled) concatenated sentences.

### Speech feature decoding

We fit the same TRF model on the same 290 principal components to predict the time course of 33 speech properties (excluding pitch derivatives, sequence statistics and stress) from population activity. We smoothed the feature time course using a 50 ms Gaussian kernel, which matches the kernel size of the PSTH. We used time delays from −300 to 100 ms and a regularization *λ* parameter of 1 × 10^8^. We shuffled the order of all sentences, fit the model on 80% of trials and evaluated accuracy on the held-out 20%. Performance was quantified using Pearson correlation between the true feature time course and the predicted feature time course.

### Reporting summary

Further information on research design is available in the Nature Portfolio Reporting Summary linked to this article.

## Online content

Any methods, additional references, Nature Portfolio reporting summaries, source data, extended data, supplementary information, acknowledgements, peer review information; details of author contributions and competing interests; and statements of data and code availability are available at 10.1038/s41586-023-06839-2.

### Supplementary information

Supplementary Information

Reporting Summary

Supplementary Video 1

Supplementary Video 2

## Data Availability

Data for which patients have consented to public release will be made available at the Data Archive for the BRAIN Initiative (DABI; https://dabi.loni.usc.edu).

## Code availability

The analysis and data visualization code will be made available on GitHub (https://github.com/ChangLabUcsf/LeonardGwilliams2023) upon publication.
