# Supplementary material for: Large-scale single-neuron speech sound encoding across the depth of human cortex
Source: Nature. 2023 Dec 13;626(7999):593–602. doi: 10.1038/s41586-023-06839-2 (PMC10866713; doi:10.1038/s41586-023-06839-2)
Supplement: Supplementary file 1 — Supplementary Information [file 41586_2023_6839_MOESM1_ESM.docx]

**SUPPLEMENTARY INFORMATION**

**Large-scale single neuron speech sound encoding across the depth of human cortex**

Matthew K. Leonard*^1,2^, Laura Gwilliams*^1,2^, Kristin K. Sellers^1,2^, Jason E. Chung^1,2^, Duo Xu^1,2^, Gavin Mischler^3,4^, Nima Mesgarani^3,4^, Marleen Welkenhuysen^5^, Barundeb Dutta^5^, & Edward F. Chang^1,2#^

^1^Department of Neurological Surgery, University of California, San Francisco, San Francisco, CA 94143, USA

^2^Weill Institute for Neurosciences, University of California, San Francisco, San Francisco, CA 94143, USA

^3^Mortimer B. Zuckerman Mind Brain Behavior, Columbia University, New York, United States

^4^Department of Electrical Engineering, Columbia University, New York, United States

^5^IMEC, Leuven, Belgium

**SUPPLEMENTARY TEXT**

**Stimulus Spectrogram Reconstruction**

To further specify how heterogeneity relates to stimulus representations and differs across sites, we decoded acoustic spectrograms from each site separately and all sites combined. We found that decoding performance was relatively high for all sites combined (reconstruction accuracy mean r=0.82*±*0.045), and while lower for individual sites (mean r range = 0.35-0.62; overall mean=0.54*±*0.08), was significantly above chance (shuffled models t=7.0; p=4e-7; **Extended Data Fig. 6a-b**). Furthermore, spectro-temporal information decoded from different sites was moderately correlated (up to r=0.3). This suggests that representations are partially shared across different sites, while the large gain in explanatory power from their combination suggests they also contain complementary information (**Extended Data Fig. 6c-d**, **Fig. 3d**).

**Population State-space Dynamics**

To understand population dynamics, we applied principal component analysis (PCA) to 623 single neurons. We found that 90% of the total variance could be explained with less than half the full dimensionality of the data, suggesting that the population representations are substantially lower dimensional than the total number of neurons (**Extended Data Fig. 8a**; 290 components = 90% variance). In addition, we found that beyond the first ~20 PCs, the contribution of more PCs was substantially less (elbow method). We next asked whether there exists a neural state-space that captures shared variance across different sentences (**Extended Data Fig. 8b**). We found that the primary speech dynamics of distinct sentences was captured by the first three PCs (PC1 Pearson r-value across 10 sentences mean=0.78±0.17; PC2 mean=0.88±0.08; PC3 mean=0.65±0.24). Within the population activity (all PCs), we were able to decode all defined speech features significantly above chance (above 95% of shuffled models; **Extended Data Fig. 8c**).

**SUPPLEMENTARY FIGURES**

**
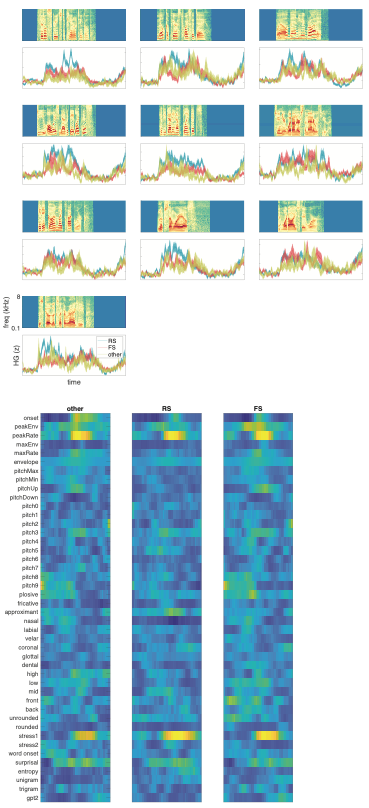
**

**SI Fig. 1**: **Average responses and TRF weights from clustering of spike waveforms into putative cell types.** Top: Average (±s.e.m.) speech-evoked responses for each of the three putative cell types in **Fig. 1g**. RS neurons tend to have higher overall firing rates, but there are no systematic differences in the dynamics of responses for each cell type. Bottom: Average TRF weights (similar to **Fig. 3**) for each putative cell type show highly similar tuning.

**
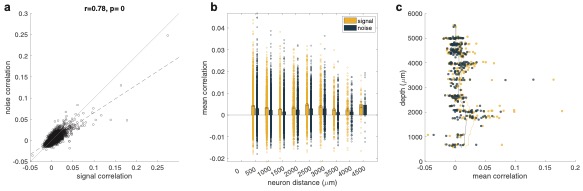
**

**SI Fig. 2: Noise and signal correlations.** **a:** Relationship between signal and noise correlations for all neuron pairs in p1. Signal and noise correlations are significantly correlated for all 9 recording sites (Pearson r=0.40-0.96, all p<10^-10^, two-sided, *n* = number of neurons per recording, see **Fig. 1e**). **b.** Signal and noise correlations as a function of distance between neuron pairs in p1 (mean±s.e.m.). Except for the most distant pairs, signal correlations were higher than noise correlations. This was difficult to assess on all recording sites due to non-homogeneous sampling of neurons across the full cortical depth in every case, however this trend was generally true in most cases. **c.** Signal and noise correlations as a function of depth. Only neuron pairs less than or equal to 100μm apart were included, and the mean depth for the pair is shown on the y-axis. For all participants including the example shown here (p1), there was no clear relationship between correlation and depth.

**SI Fig. 3: dSTRF hierarchical clustering.** Unsupervised hierarchical clustering (minimum-variance, Euclidean distance) on the three non-linearities reveals two primary clusters of neurons (**Fig. 5l-m**).

**SI Fig. 4: Individual spike waveforms for each putative cell type.** Clustering of single neuron spike waveforms (**Fig. 1g**) produces three distinct waveform shapes, which are consistent for all neurons in each cluster.

**SUPPLEMENTARY MOVIES**

**Supplementary Video 1: All 685 neurons recorded from human STG.** Individual sentences (spectrograms; top) with single trial rasters for all neurons (middle). The average across all 685 neurons is shown as a normalized PSTH (bottom). The sound intensity and pitch corresponds to the amplitude of the PSTH.

**Supplementary Video 2: Example single trial rasters for one speech-responsive neuron.** Spikes in response to all ten repetitions of the same sentence for a single neuron (p4-2 u79). Spike times are highly similar across repetitions, and tend to occur in response to nasal sounds like /n/ and /m/.

**SUPPLEMENTARY TABLES**

**SI Table 1: Feature descriptions**

| **Feature** | **Class** | **Description** |
| --- | --- | --- |
| Sentence onset | Onset | Binary impulse at the start of each sentence |
| Envelope | Intensity | Continuous analytic amplitude envelope of speech, square rectified and filtered from 1-10Hz |
| peakEnv | Intensity | Local peaks in the continuous envelope, code as a sparse impulse where the magnitude of the impulse corresponds to the magnitude of the peak |
| maxEnv | Intensity | Binary impulse at the maximum peakEnv event for each sentence |
| peakRate | Intensity | Local peaks in the positive derivative of the continuous envelope, where the magnitude of the impulse corresponds to the slope of the envelope derivative |
| maxRate | Intensity | Binary impulse at the maximum peakRate event for each sentence |
| Pitch | Pitch | Relative vocal pitch (normalized within each sentence), binned into ten equally-spaced groups. The feature is defined for the duration with which the pitch track stays within the given percentile range. |
| pitchUp | Pitch | Local peaks in the positive derivative of the continuous pitch track |
| pitchDown | Pitch | Local peaks in the negative derivative of the continuous pitch track |
| maxPitch | Pitch | Binary impulse at the maximum pitch for each sentence |
| minPitch | Pitch | Binary impulse at the minimum pitch for each sentence |
| Plosive | Consonant | Binary impulse at the onset of each phoneme with a plosive manner of articulation |
| Approximant | Consonant | Binary impulse at the onset of each phoneme with an approximant manner of articulation |
| Fricative | Consonant | Binary impulse at the onset of each phoneme with a fricative manner of articulation |
| Nasal | Consonant | Binary impulse at the onset of each phoneme with a nasal manner of articulation |
| Labial | Consonant | Binary impulse at the onset of each phoneme with a labial place of articulation |
| Velar | Consonant | Binary impulse at the onset of each phoneme with a velar place of articulation |
| Coronal | Consonant | Binary impulse at the onset of each phoneme with a coronal place of articulation |
| Glottal | Consonant | Binary impulse at the onset of each phoneme with a glottal place of articulation |
| Dental | Consonant | Binary impulse at the onset of each phoneme with a dental place of articulation |
| High | Vowel | Binary impulse at the onset of each phoneme with a high vowel place of articulation |
| Low | Vowel | Binary impulse at the onset of each phoneme with a low vowel place of articulation |
| Mid | Vowel | Binary impulse at the onset of each phoneme with a mid vowel place of articulation |
| Front | Vowel | Binary impulse at the onset of each phoneme with a front vowel place of articulation |
| Back | Vowel | Binary impulse at the onset of each phoneme with a back vowel place of articulation |
| Rounded | Vowel | Binary impulse at the onset of each phoneme with a round vowel place of articulation |
| Unrounded | Vowel | Binary impulse at the onset of each phoneme with an unrounded vowel place of articulation |
| Primary stress | Stress | Binary impulse at the onset of each syllable with primary lexical stress |
| Secondary stress | Stress | Binary impulse at the onset of each syllable with secondary lexical stress |
| Word onset | Stats | Binary impulse at the onset of each word |
| Phoneme surprisal | Sequence probability | Impulse at the onset of each phoneme, where the magnitude is the surprisal value of each phoneme given all prior phonemes in the word |
| Phoneme entropy | Sequence probability | Impulse at the onset of each phoneme, where the magnitude is the phoneme entropy of each phoneme given all prior phonemes in the word |
| Unigram surprisal | Sequence probability | Impulse at the onset of each word, where the magnitude is proportional to surprisal value as computed using a unigram model (i.e. equivalent to word frequency) |
| Trigram surprisal | Sequence probability | Impulse at the onset of each word, where the magnitude is proportional to surprisal value as computed using a trigram model |
| GPT2 surprisal | Sequence probability | Impulse at the onset of each word, where the magnitude is proportional to surprisal value as computed using GPT2 |

**SI** **Table 2: Participant characteristics**

|  | **Sex** | **Handedness** | **Hemisphere** | **Lesional/Non-lesional** | **Lesion Pathology** | **ECoG/Seizure Focus** | **Surgical Plan** |
| --- | --- | --- | --- | --- | --- | --- | --- |
| p1 | F | R | L | non-lesional | HS+cortical gliosis | grid: medial and lateral temporal regional onset | tailored temporal lobectomy |
| p2 | M | R | L | non-lesional | HS + cortical gliosis | grid: medial onset | extended ATL |
| p3 | M | R | L | non-lesional | encephalitis | Grid: lateral temporal onset in posterior temporal lobe, refractory to RNS therapy | lateral temporal lobe |
| p4 | F | R | R | non-lesional | focal cortical dysplasia type 2A | grid: lateral temporal and parietal cortex | tailored temporoparietal resection |
| p5 | F | R | L | mesial temporal sclerosis | HS + cortical gliosis | No grid | ATL- anterior resection |
| p6 | M | R | L | brain tumor | brain metastasis | N/A | temporal resection |
| p7 | M | R | L | non-lesional | mild gliosis in cortex, moderate HS | grid: medial onset | ATL |
| p8 | M | R | L | mesial temporal sclerosis | gliosis | No grid | ATL |

HS: hippocampal sclerosis; FCD: focal cortical dysplasia; ATL: anterior temporal lobectomy;
